# Supplementary figures and images for: Comprehensive Genomic and Phenotypic Characterization of Escherichia coli O78:H9 Strain HPVN24 Isolated from Diarrheic Poultry in Vietnam
Source: Microorganisms. 2025 Sep 26;13(10):2265. doi: 10.3390/microorganisms13102265 (PMC12565876; doi:10.3390/microorganisms13102265)

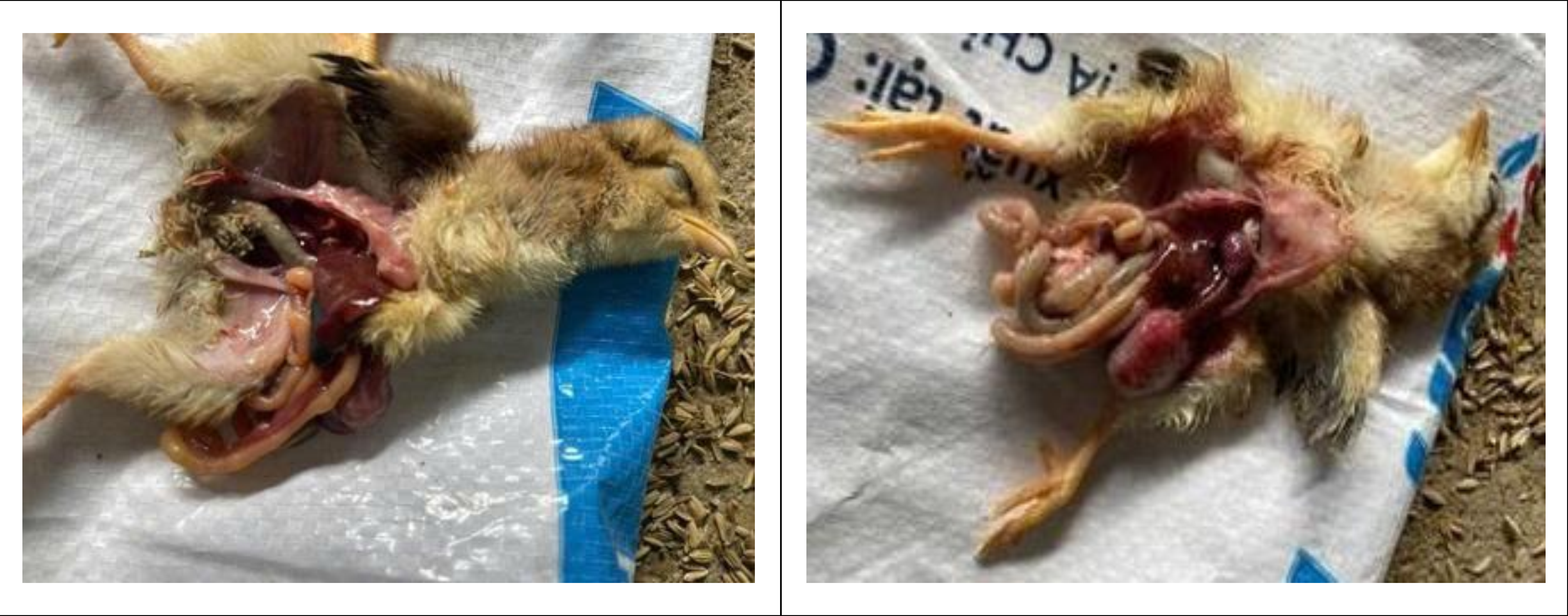

Supplement: Supplementary file 1 [file microorganisms-13-02265-s001.zip › Supplementary Files/Figure S1.png]

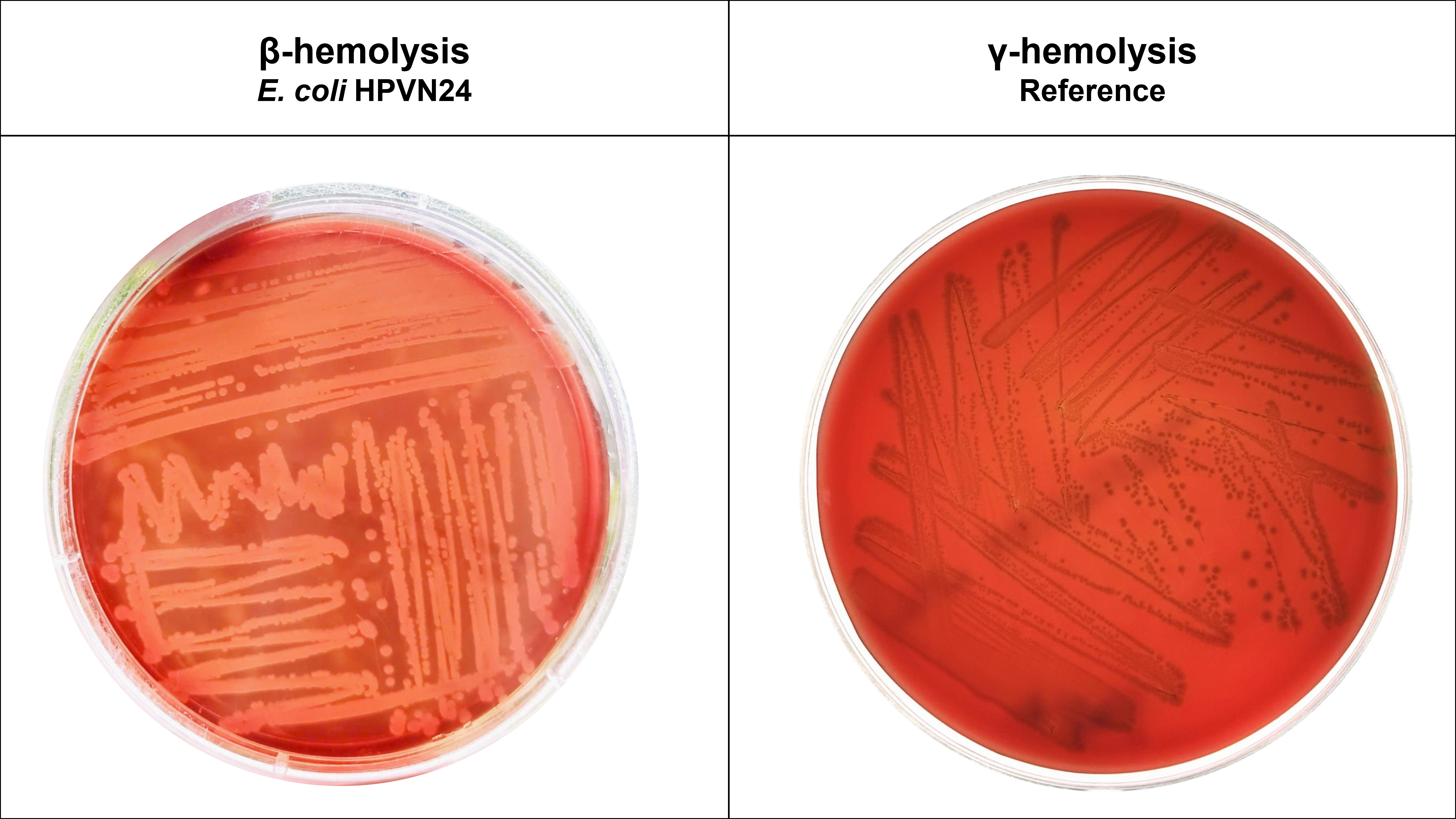

Supplement: Supplementary file 1 [file microorganisms-13-02265-s001.zip › Supplementary Files/Figure S2.png]

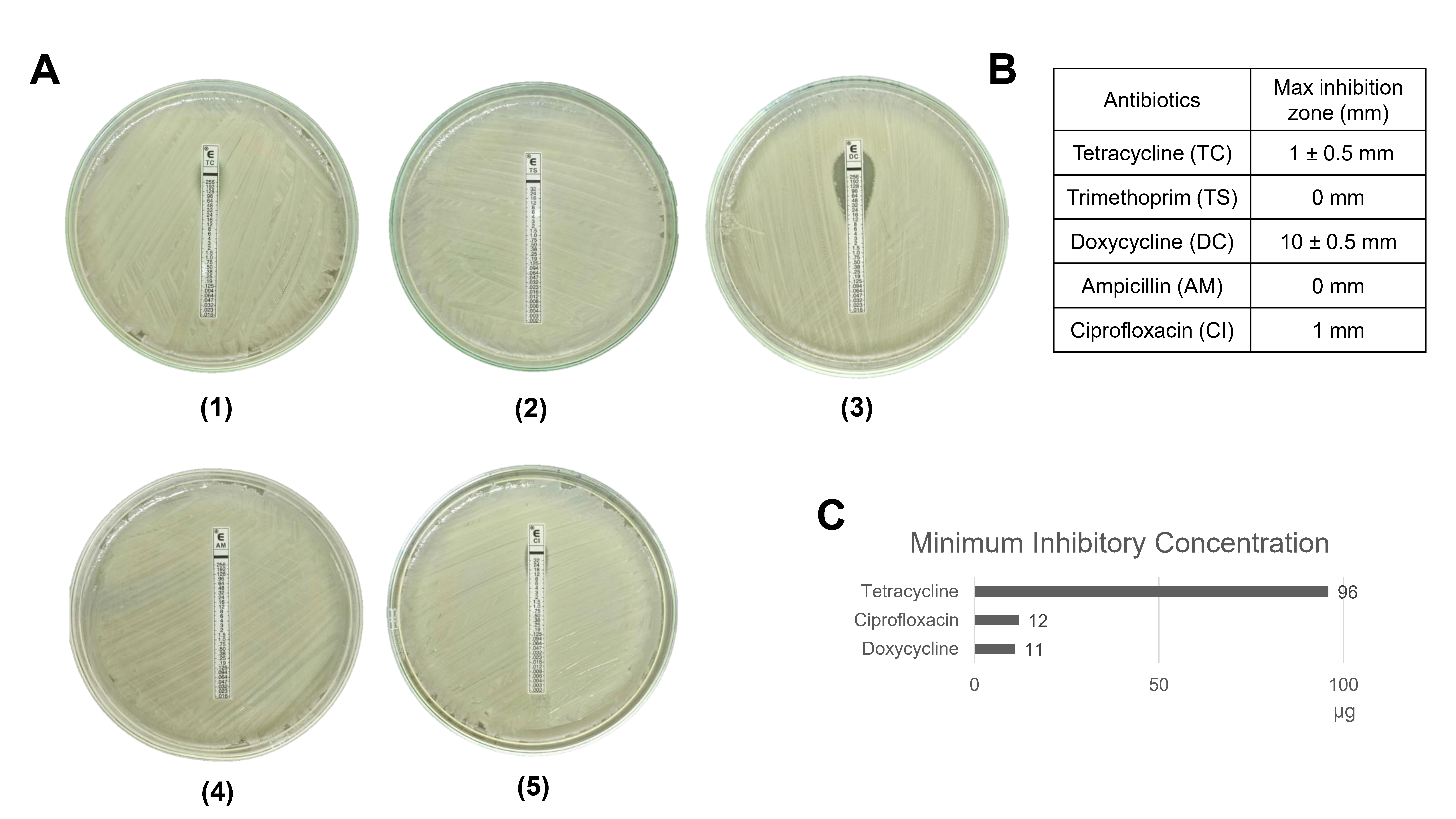

Supplement: Supplementary file 1 [file microorganisms-13-02265-s001.zip › Supplementary Files/Figure S3.png]
